# Exploring Crystal Structure Features in Proton Exchange Membranes and Their Correlation with Proton and Heat Transport

**DOI:** 10.3390/polym16233250

**Published:** 2024-11-22

**Authors:** Cong Feng, Cong Luo, Pingwen Ming, Cunman Zhang

**Affiliations:** 1 College of Materials Science and Engineering, Tongji University, Shanghai 201804, China; 2130588@tongji.edu.cn; 2 School of Automotive Studies, Tongji University, Shanghai 201804, China; pwming@tongji.edu.cn (P.M.); zhangcunman@tongji.edu.cn (C.Z.)

**Keywords:** semicrystalline structure, multiscale simulations, proton conductivity, thermal properties, characterization tests

## Abstract

Proton exchange membranes (PEMs) are dominated by semicrystalline structures because highly pure crystals are still challenging to produce and control. Currently, the development and application of PEMs have been hindered by a lack of understanding regarding the effects of microstructure on proton and heat transport properties. Based on an experimentally characterized perfluoro sulfonic acid membrane, the corresponding semicrystalline model and the crystal model contained therein were constructed. The water distribution, proton, and heat transport in the crystal, amorphous, and semicrystalline regions were examined using molecular dynamics simulations and energy-conserving dissipative particle dynamics simulations. The crystal structure had pronounced water connection pathways, a proton transport efficiency 5–10 times higher than that of the amorphous structure, and an in-plane covalent bonding that boosted the thermal diffusion coefficient and thermal conductivity by more than 1–3 times. The results for the semicrystalline structure were validated by the corresponding experiments. In addition, a proportionality coefficient that depended on both temperature and water content was proposed to explain how vehicle transport contributed to the proton conductivities, facilitating our understanding of the proton transport mechanism. Our findings enhance our theoretical understanding of PEMs in proton and heat transport, considering both the semicrystalline and crystalline regions. Additionally, the research methods employed can be applied to the study of other semicrystalline polymers.

## 1. Introduction

Proton exchange membranes (PEMs), with their potential to address pressing energy and environmental challenges, are critical components of fuel cells and water electrolysis systems [1,2,3,4]. Despite significant advances in research, development, and practical applications, the widespread commercialization of PEMs remains hindered by various technical and economic obstacles. Among the primary concerns are the challenges of balancing chemical and thermal stability, high proton conductivity, and cost-effectiveness [5,6,7]. Currently, perfluorosulfonic acid (PFSA) is the dominant material used in commercial PEMs, with newly developed membranes often being compared with the performance of Nafion, a widely-used PFSA-based membrane. Consequently, understanding the nanostructure of Nafion and its correlation with its properties is crucial for evaluating novel PEM materials. However, the molecular-level nanostructure of Nafion and its conductive properties remain areas of uncertainty. Specifically, the configuration, distribution, and volume occupancy of crystal regions within Nafion and their effects on proton conduction and thermal properties are critical factors influencing PEM functionality.

In recent years, single-crystal proton-conducting materials, such as metal–organic frameworks (MOFs) [8,9,10], hydrogen-bonded organic frameworks [11,12], and polyoxometalates (POMs) [13], have garnered considerable attention due to their well-defined structures and distinguishable proton transmission pathways. For example, the POM crystal material discovered by Wang et al. exhibited high proton conductivity, reaching 0.19 S/cm along the [001] direction at 85 °C and 98% relative humidity. When processed into a composite membrane, it demonstrated peak proton conductivity of 5.31 × 10^−2^ S/cm [13]. These findings suggest that the conductivity of the crystal segments within PEMs significantly influences their overall conductivity. Similarly, Nafion membranes, with their semicrystalline nanostructure, typically achieve proton conductivities of 0.1 S/cm under comparable temperature and humidity conditions. This raises the question of how increasing the grain size and crystallinity of Nafion membranes might affect proton conductivity.

Studies on the microstructures of Nafion have been conducted using small-angle X-ray scattering (SAXS), wide-angle X-ray scattering (WAXS), and X-ray diffraction (XRD). The SAXS profile reveals two peaks: one corresponding to ionic clusters and the other to the hydrophobic matrix [14,15]. The first peak is attributed to ionic clusters, while the second is associated with lamellar tetrafluoroethylene crystallites. In the WAXS plots, the observed peak indicates the crystalline phase, and both crystallite size and crystallinity generally increase with heating cycles from 40 to 250 °C [16]. However, the crystallinity of Nafion can vary depending on the preparation method, fabrication process, and testing techniques. For example, the crystallinity of Nafion with an equivalent weight (EW) of 1100 was 23% in its nonionic form and 18–14% in its carboxylated form, with crystallinity increasing as the EW value increased [17]. For the untreated commercial Nafion 117 membrane, XRD measurements typically showed a crystallinity between 27% and 30% [18,19].

Simulation is another effective method of investigating the microstructural characteristics of Nafion membranes. For example, all-atom molecular dynamics simulations, due to molecular vibrations and transport occurring on a picosecond timescale, offer significant advantages in studying the structural characteristics and dynamic properties of PFSA membranes [20,21,22]. Short side-chain ionomers, such as Aquivion, exhibit higher diffusion coefficients for water molecules and hydronium ions compared with Nafion systems [21]. This is attributed to the shorter side chains, which facilitate the movement of hydronium ions among host groups, enhancing chain adsorption [22]. Dissipative particle dynamics simulation has the advantage of reconstructing the water-phase structure and analyzing the diffusion of particles and tortuosity of the hydrated membrane at a mesoscopic scale [23,24,25]. Furthermore, two-dimensional pattern simulations, through calculated electron density maps, can approximate SAXS curves, optimizing the distribution and characteristics of crystalline, semicrystalline, and amorphous regions, thus explaining the structural features of Nafion [26]. While these investigations provide valuable insights into the nanostructure and its associated particle transport properties, the atomistic arrangement in crystalline regions and their contribution to particle transport and heat transport have been less reported.

In addition to proton conduction, efficient heat transfer is another essential property of PEMs, as thermal management is crucial for the performance of PEM fuel cell (PEMFC) stacks [27,28]. Various studies have focused on improving system structures, enhancing heat transfer efficiency, and boosting the thermal properties of key materials [29,30,31]. Early investigations have shown that the thermal conductivity of Nafion ranges from 0.2 to 0.4 W/(m·K), increasing with water content and varying with temperature [32,33,34,35,36]. However, the thermal properties of single-crystal or semicrystalline PEM materials differ significantly from their amorphous counterparts due to in-plane covalent bonding. Factors such as phonon collision probability, transmission, and diffusivity—crucial for heat transfer—are notably different between these material phases [37,38,39].

This study aims to determine the nanostructural characteristics of PEM materials, particularly the crystal regions, such as lattice structure, grain size, and volume occupancy ratio. Through a combination of experimental and simulation methods, we seek to establish a relationship between the semicrystalline microstructure of PEMs and their macroscopic properties related to proton and heat transport.

## 2. Research Methods

### 2.1. Microstructure Characterization

The semicrystalline morphology of Nafion was characterized using high-resolution transmission electron microscopy (HR-TEM) (JEM-2100F, JEOL, Tokyo, Japan). A Nafion D2020 resin solution with an initial concentration of 20 wt% was diluted to 1 wt% using isopropyl alcohol to prepare the sample. The crystal characteristics of the commercial Nafion 117 membrane were then analyzed via XRD (DX-2700BH, HAOYUAN, Fuyang, China) at room temperature.

**Figure 1:**
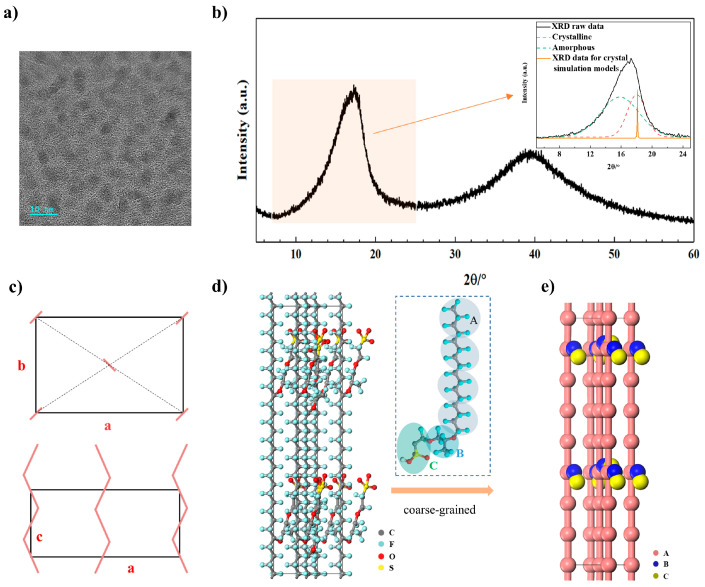
Characterization and modeling of the crystal structure. (**a**) HR-TEM and (**b**) XRD patterns of Nafion 117, and the inset shows the crystal and amorphous peaks of XRD, as well as the crystal peak of molecular modeling. (**c**) Schematic representative, (**d**) all-atom model, and (**e**) coarse-grained model of the crystal structure.

The HR-TEM image of Nafion’s semicrystalline nanostructure is presented in Figure 1a, where crystal regions (dark zones) appear as approximately 4 nm circular spots, randomly dispersed within the amorphous matrix. The XRD pattern for Nafion 117 (Figure 1b) showed a sharp peak at 2*θ* = 17.89°, corresponding to an interplanar crystal spacing of *d* = 4.95 Å. These data suggest a crystallinity of 28% and a grain size of 4.41 nm, consistent with findings in the literature [18,19,40]. A second broad peak around 2*θ* = 38° was attributed to intra-chain spacing, likely resulting from overlapping diffraction along the polymer chain axis due to intramolecular correlations in both amorphous and crystal phases [41,42].

### 2.2. Modeling and Validation of Crystal Structure

By integrating the XRD data and HR-TEM images, it was deduced that Nafion possesses an orthorhombic crystal structure with unit cell dimensions of *a* = 9.9 Å, *b* = 5.6 Å and *c* = 2.8 Å (Figure 1c). These results align with those reported by Heijden et al. [42]. Using this information, an all-atom molecular model was constructed to represent the crystal structure (Figure 1d), and the XRD intensity for this model was calculated. The calculated XRD results closely matched the experimental Nafion XRD pattern (inset in Figure 1b), particularly at the peak corresponding to the (2 0 0) crystal plane, confirming the accuracy of the model.

Since the real membrane scale is much larger than that of the all-atom model, a coarse-grained model was developed to extend the simulation scales. In the coarse-grained model, each particle represents a collection of atoms. For instance, four water molecules are grouped into a bead (W), with the radius *R_c_* = 7.82 Å, derived from water’s mass and density. Three hydrated protons form a bead (P). The Nafion backbone is divided into beads (A) comprising 12 atoms (-CF2-CF2-CF2-CF2-), while the side chain is represented by bead B (-O-CF3-CF-CF2-O-) and bead C (-CF2-CF2-SO3-) (Figure 1e).

### 2.3. Semicrystalline and Amorphous Structures

For semicrystalline structures, larger coarse-grained models were constructed to encompass multiple crystal and amorphous regions. Crystal models were randomly placed in a simulation box, followed by the addition of amorphous polymer chains to match the observed crystallinity of 28%. This semicrystalline unit cell (Figure S1) was then enlarged by a factor of three along the x, y, and z axes, resulting in a final model size of 30–40 nm.

Amorphous structures were created by randomly arranging polymer chains in a periodic box. The size of these boxes and the number of molecular chains were kept identical to those used in the crystal structures, ensuring that the comparison focused solely on structural effects without introducing other variables.

### 2.4. Simulation Details

To investigate proton transport and thermal properties, energy-conserving dissipative particle dynamics (eDPDs) and all-atom molecular dynamics (AAMDs) simulations were employed. These simulations were conducted using the large-scale atomic/molecular massively parallel simulator (LAMMPS) [43]. The two simulation methods complement each other, as eDPDs enable analysis on larger time and size scales, while AAMDs provide detailed insights into atomic-level interactions in both crystal and amorphous regions.

In the AAMD simulations, interactions between water molecules were modeled using the TIP3P water model, while the perfluorosulfonic acid system was described using the DREIDING force field. This force field was validated by comparing calculated energy values with those obtained from the commercial software Materials Studio 6.0. Specific parameters and validation results are provided in Tables S1–S5. For eDPD simulations, derived parameters and equations are detailed in the Supplementary Material (Note S3).

Periodic boundary conditions were applied to all models. To investigate proton transport under the vehicle mechanism, a constant electric field of 1 × 10^3^ V/m was applied along one direction of the PEM model, simulating the potential difference typical of PEMFC conditions. The hydronium ion diffusion coefficient (D) was calculated using Einstein’s diffusion law, based on the mean square displacement (MSD) curves (Figure S2a). Proton conductivity ($\sigma_{V}$) was then derived from the diffusion coefficient [44]. Heat transport simulations were performed along the Z-axis of the models. Heat was applied to the top and removed from the bottom of the simulation box over 50,000 time steps to reach thermal equilibrium (Figure S2b). The final 20,000 steps were used to monitor temperature gradients across 1 nm slices along the Z-axis (Figure S2c).

### 2.5. Thermal Performance Testing

The thermal properties of Nafion 117, including thermal diffusivity and thermal conductivity, were experimentally measured to validate the simulation results. The thermal diffusivity was determined using a laser thermal conductivity instrument (LFA 467, HyperFlash, NETZSCH, Sable, Germany). Four identical samples were tested, with each sample coated with carbon to enhance measurement accuracy. Thermal conductivity measurements were conducted using a thermal conductivity analyzer (TPS3500, Hot Disk, Uppsala, Sweden), which calculates conductivity by monitoring voltage shifts during heat transfer. Three samples were tested at each temperature to ensure the reproducibility and consistency of the data.

## 3. Results and Discussion

### 3.1. Water Distribution

Understanding water distribution within the proton exchange membrane (PEM) is essential for analyzing both proton and heat transport mechanisms. In this study, water distribution was examined under three hydration levels (denoted as *λ* = 3, 11, and 21) across a temperature range of 300 to 360 K, reflecting typical operating conditions of fuel cells. The water distribution patterns within both the crystal and amorphous structures were simulated using AAMD and eDPD, showing consistent results (Figure S3).

**Figure 2:**
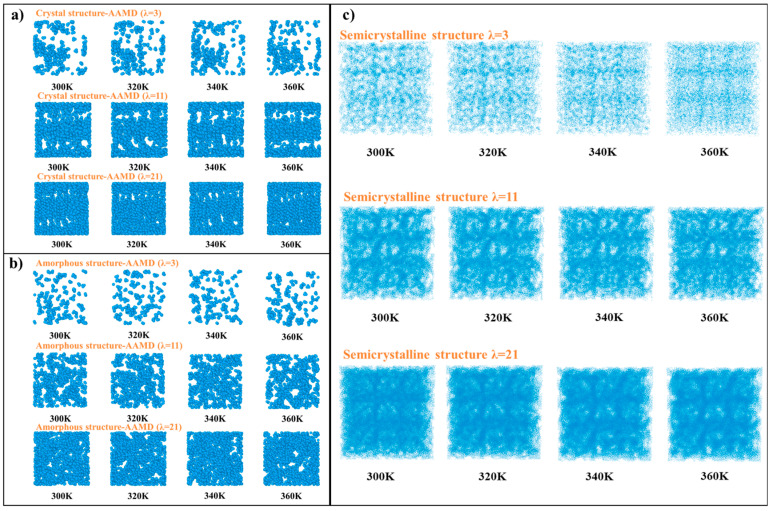
Distribution of water molecules in (**a**) crystal, (**b**) amorphous, and (**c**) semicrystalline structures.

The simulations revealed that the morphology of water channels was influenced by both temperature and hydration levels. As shown in Figure 2a, in the crystal regions, a layered water distribution was observed, where water molecules were concentrated between adjacent polymer backbone chains. This layering effect arose from the regular arrangement of hydrophilic groups within the crystal domains, which promoted the formation of connected water channels adjacent to the ionomer side chains. However, at the lowest hydration level (*λ* = 3), the structure lacked pronounced layering due to insufficient water content. As the water content increased (*λ* = 11), a partially delaminated structure emerged, while at the highest hydration level (*λ* = 21), the structure became more well-defined and ordered.

In contrast, the water distribution within amorphous regions showed no specific regular structure (Figure 2b). Water molecules were more randomly dispersed, lacking the organization seen in the crystal regions. Nevertheless, the semicrystalline structure, which contained both crystal and amorphous domains, exhibited a more ordered water distribution than the fully amorphous structure (Figure 2c). This was because the crystal regions helped to organize the water channels. Interestingly, temperature appeared to have a lesser impact on water distribution than hydration level, as the water molecules were primarily influenced by structural arrangements, rather than thermal fluctuations.

### 3.2. Proton-Conducting Properties

#### 3.2.1. Influence Factors

**Figure 3:**
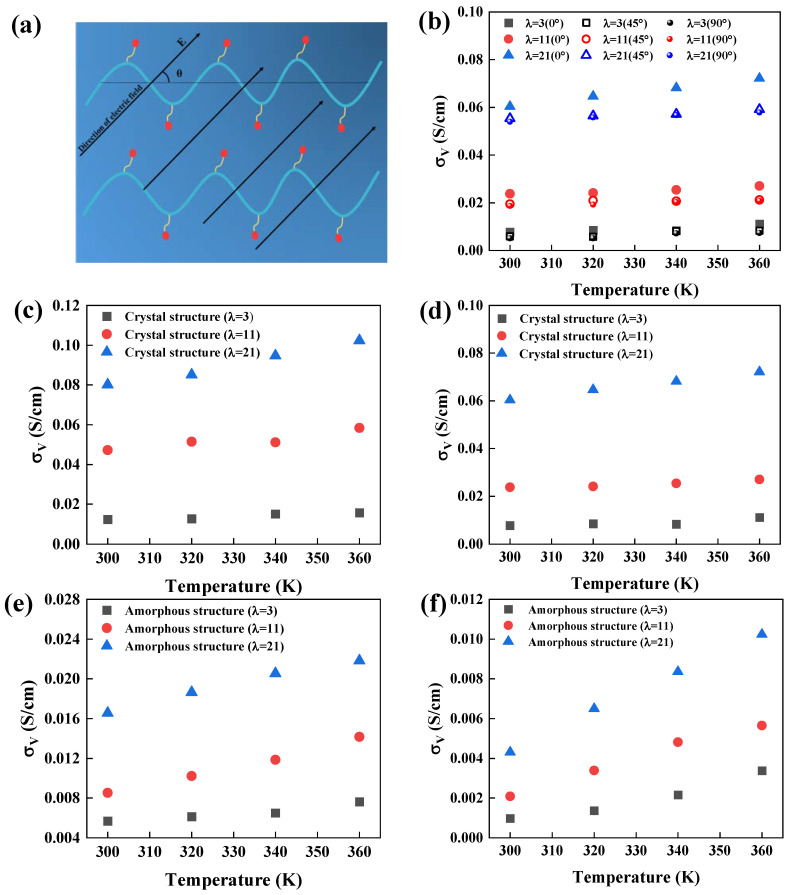
(**a**) Schematic of the direction of the electric field and the structure of the backbone chain arrangement. (**b**) Proton conductivity in different electric field directions. (**c**–**f**) Proton conductivity in crystal and amorphous structures from AAMD and eDPD simulations.

The parameter $\sigma_{V}$, which is governed by the vehicle transport mechanism of PEMs, was investigated in detail. Given the anisotropic nature of crystal structures, special attention was paid to how proton conductivity changes with respect to the orientation of the electric field relative to the polymer backbone. Figure 3a illustrates this setup, where *θ* = 0° represents a field direction parallel to the backbone chain.

In crystal structures, proton transport is facilitated by the presence of continuous hydrophilic channels running parallel to the backbone. When the electric field is applied along this direction (*θ* = 0°), proton conductivity is highest. However, as the field angle increases, conductivity decreases significantly (Figure 3b). Proton transport is least efficient when the electric field is applied perpendicular to the proton-conducting channels (*θ* = 90°), but even under these conditions, proton conductivity in the crystal regions is still notably higher than that of amorphous regions. This highlights the critical role that regularly arranged crystal structures play in enhancing proton transport.

The simulations (Figure 3c,d) further demonstrated that crystal structures exhibited proton conductivities that are five to ten times higher than those of amorphous structures. Both temperature and water content positively influenced conductivity by increasing the fluidity of the water channels and the thermal motion of atoms, thereby accelerating proton transport. In contrast, in amorphous regions, the entangled polymer chains obstructed the water channels, leading to slower proton transport. Additionally, the proton conductivity from eDPD simulations was slightly higher than that obtained from AAMD simulations, likely due to the larger time and system scales accessible in eDPD (Figure 3e,f). Semicrystalline structures, owing to their mixed composition, exhibited intermediate conductivity between fully crystal and amorphous structures (Figure S4). As a result, the proton conductivity exhibited a progressive decrease in crystal, semicrystalline, and amorphous structures, and were primarily determined by crystallinity, with less influence from other factors like temperature, water content, and electric field direction.

#### 3.2.2. Relationship Between Microstructure and Macroscopic Properties

**Figure 4:**
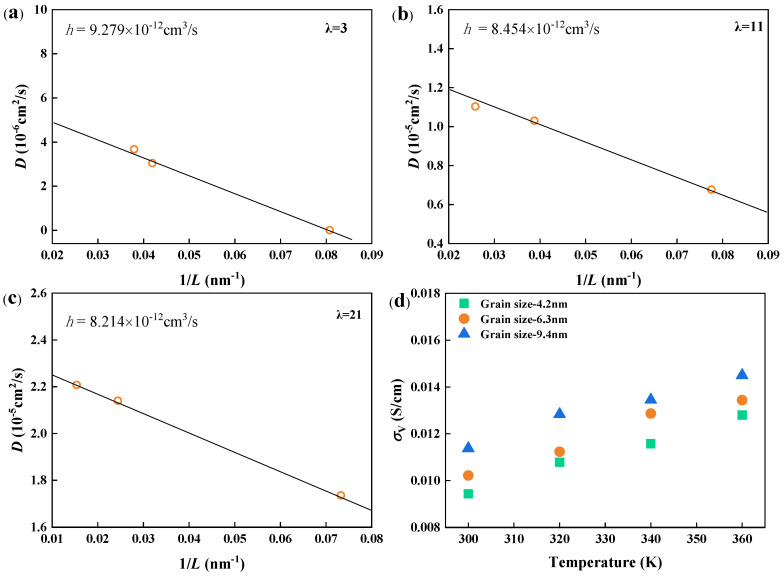
Proton diffusion coefficients of semicrystalline structures as a function of 1/L at (**a**) λ = 3, (**b**) λ = 11, and (**c**) λ = 21. (**d**) Proton conductivity in the same semicrystalline structure with different grain sizes.

Controlling the macroscopic properties of PEMs through their microstructural design is crucial for optimizing proton conductivity. It is well-known that periodic boundary conditions (PBCs) in molecular simulations can introduce boundary effects, leading to deviations in particle transport compared to macroscopic models. To address this, an empirical relationship was applied to calculate the correlation of the diffusion coefficient under PBC conditions (*D_PBC_*) with the diffusion coefficient in an infinite system (*D*_0_) using the equation *D_PBC_* = *D_0_* − *h*/*LD_PBC_*, where *L* is the box length and *h* is a constant [45,46,47,48]. This approach allowed us to account for system size effects and calculate proton conductivity more accurately for semicrystalline structures (Figure 4a–c).

Next, the effect of grain size on proton conductivity was explored in semicrystalline structures at *λ* = 3 with a constant crystallinity of 28%. As shown in Figure 4d, larger grain sizes resulted in higher proton transport due to the improved connectivity of water channels. This suggests that increasing the crystallinity or grain size of PEM materials could enhance proton transport efficiency.

#### 3.2.3. Proportionality Coefficient $\sigma_{E}/\sigma_{V}$

Electrochemical impedance spectroscopy (EIS) was used to determine the total proton conductivity ($\sigma_{E}$) and compare it with the vehicle transport conductivity ($\sigma_{V}$). The proportionality coefficient *N* = $\sigma_{E}$/$\sigma_{V}$ was calculated, which provides insight into the relative contributions of the Vehicle and Grotthuss mechanisms to overall proton transport. Here, $\sigma_{V}$ was derived from our semicrystalline model, which represented a real Nafion membrane, as both shared similar structural properties, such as crystallinity, crystal size and structural parameters. It had the following fitted expression under different environmental conditions:

(1) $$\sigma_{V}=\left(3.906\lambda +1\right)\exp\left(-5.737-\frac{379.978}{T}\right)$$

Using the proton conductivity *σ_E_* of Nafion 117, proposed in a previous study [49] as a function of temperature (*T*) and water content (*λ*), the proportionality coefficient N can be expressed as follows

(2) $$\frac{\sigma_{E}}{\sigma_{V}}=N=\left(0.01\lambda +1\right)\exp\left(2.853-\frac{817.653}{T}\right)$$

**Figure 5:**
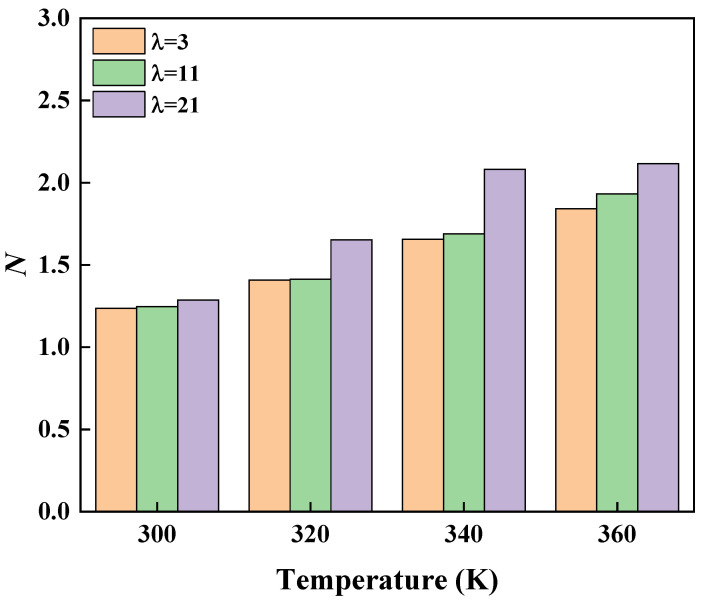
Proportionality coefficient *N* versus temperature and water content.

Figure S4 presents the specific values of $\sigma_{V}$ and $\sigma_{E}$ at different temperatures and water contents. *N* increased with both temperature and water content, as shown in Figure 5, indicating that the Grotthuss mechanism became increasingly dominant under these conditions.

### 3.3. Thermal Properties

The thermal properties of Nafion, including thermal diffusivity, specific heat capacity, and thermal conductivity, were investigated through both simulations and experiments. The simulated system included three different water contents (*λ* = 3, 11, and 21). In contrast, due to the uncontrollable humidity conditions in the chamber of the experimental apparatus, only samples with humidity levels similar to those of the surrounding atmosphere were measured, corresponding to *λ* values of 4–5. These thermal properties were primarily determined by the atomic molar heat capacity of the system, which was analyzed in detail (Figure S5). A periodic heat conduction simulation was conducted by applying a non-contact heat source to each coarse-grained particle in the eDPD model [50] (Figure S2b).

#### 3.3.1. Thermal Diffusion Coefficients

**Figure 6:**
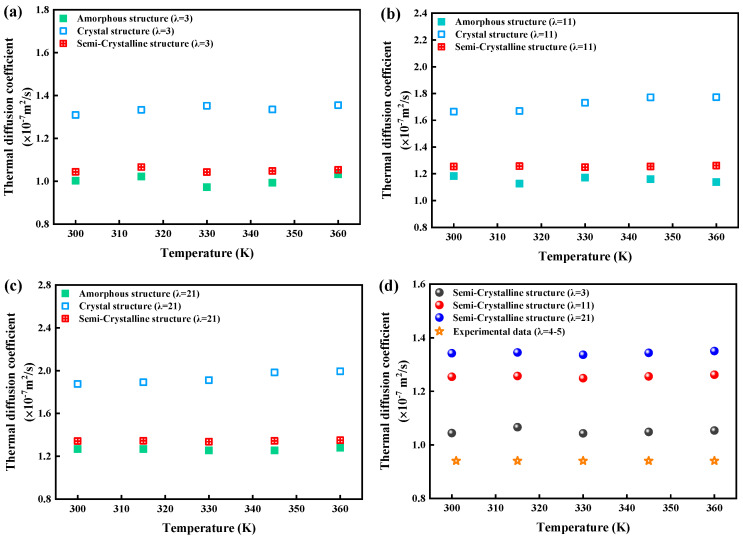
Thermal diffusion coefficient of crystal, amorphous and semicrystalline structures at (**a**) *λ* = 3, (**b**) *λ* = 11, and (**c**) *λ* = 21. (**d**) Comparison of simulation and experimental results.

The thermal diffusion coefficients for crystal, amorphous, and semicrystalline structures are presented in Figure 6a–c. In crystal structures, phonon mobility is enhanced due to the reduced probability of phonon collisions, resulting in a higher thermal diffusion coefficient compared with amorphous structures. Water, with a higher thermal diffusion coefficient than Nafion, further increased the overall thermal diffusivity as water content increased between 30 and 360 K. However, temperature had a relatively minor effect on thermal diffusion coefficients within the studied range. The comparison between the simulation and experimental results (Figure 6d) showed good agreement, though the simulated values were slightly higher, likely due to the smaller time and system scales used in the simulations.

#### 3.3.2. Specific Heat Capacity

**Figure 7:**
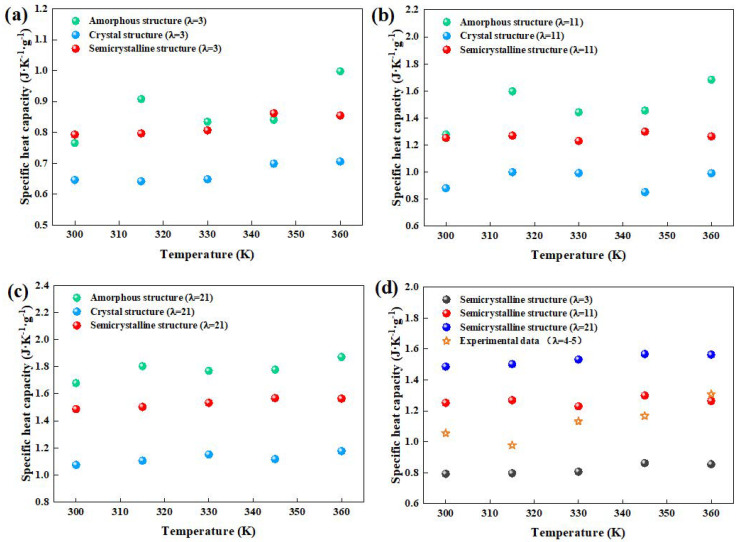
Specific heat capacity of crystal, amorphous, and semicrystalline structures at (**a**) *λ* = 3, (**b**) *λ* = 11, and (**c**) *λ* = 21. (**d**) Comparison of simulation and experimental results.

The specific heat capacities of the crystal, semicrystalline, and amorphous structures are illustrated in Figure 7. Specific heat capacity increased with both temperature and water content. Crystal structures, due to their denser atomic packing and restricted atomic vibrations, exhibited lower specific heat capacities than amorphous structures. Additionally, because water has a higher specific heat capacity than Nafion, increasing water content increased the overall heat capacity. The simulation results aligned well with the experimental data, particularly for hydration levels *λ* = 3 and *λ* = 11, as shown in Figure 7d.

#### 3.3.3. Thermal Conductivity

**Figure 8:**
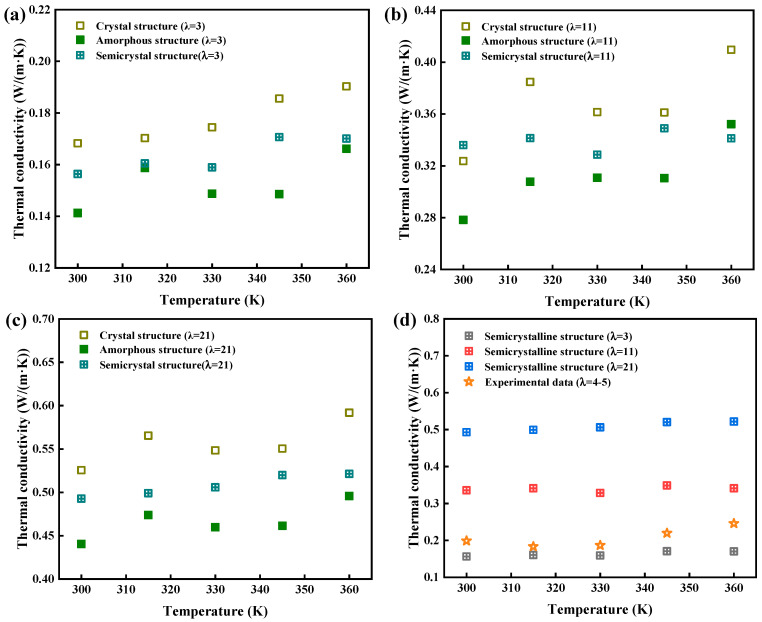
Thermal conductivity of crystal, amorphous, and semicrystalline structures, as calculated from eDPD simulations, for (**a**) λ = 3, (**b**) λ = 11, and (**c**) λ = 21; and (**d**) a comparison of Nafion membrane’s thermal conductivity, measured experimentally and calculated from eDPD simulations, for semicrystalline structures. The hollow, solid, hollow with cross, and pentagram symbols represent the thermal conductivity of the crystal, amorphous, and semicrystalline structures and Nafion 117 membrane, respectively.

Thermal conductivity (*κ*) was calculated using the relationship between thermal diffusivity (*α*), specific heat capacity (*C*), and density (*ρ*), where *κ* = *α* × *ρ* × *C*. As seen in Figure 8, thermal conductivity increased with temperature, driven by the increase in specific heat capacity, while thermal diffusivity remained relatively constant. Crystal structures had higher thermal conductivity (about 1.3 times) compared with amorphous structures due to their longer phonon mean free paths, with semicrystalline structures displaying intermediate values. The simulation results agreed well with experimental data for Nafion 117 (Figure 8d). Therefore, thermal conductivity was primarily influenced by the structural organization and water content of the material, with crystal structures outperforming amorphous regions. These results provide valuable insights into the thermal management of PEMs in fuel cell applications.

## 4. Conclusions

In conclusion, molecular simulations and experimental characterizations were employed to establish and validate a crystal structure model of the Nafion membrane. The regular arrangement of side chains in the crystal structure facilitated a smoother and more connected distribution of water molecules, enabling efficient proton transport with minimal dependence on the direction of the electric field. This behavior contrasted with that observed in amorphous and semicrystalline structures. Specifically, for a given crystallinity and environmental conditions, an increase in grain size led to enhanced proton conductivity. The diffusion coefficient was extrapolated from periodic boundary systems to macroscopic systems using a scaling factor *h*, and the contribution of vehicle transport to proton conductivity was quantified through the proportionality coefficient *N*.

Additionally, thermal properties, such as the thermal diffusion coefficient, specific heat capacity, and thermal conductivity of PEMs, were determined and confirmed through experimentation. At the same temperature and water content, the crystal structure exhibited the highest thermal conductivity and diffusion coefficient, while the amorphous structure exhibited the lowest. This study provides valuable insights into the relationship between the microstructural features of semicrystalline PEMs and their proton and heat transport properties. These findings will not only contribute to the development of novel PEM materials and inform technical applications based on molecular interaction mechanisms, but also serve as a methodological reference for exploring other semicrystalline polymer materials.

## Data Availability

The original contributions presented in the study are included in the article/Supplementary Materials, further inquiries can be directed to the corresponding author.

## Supplementary Materials

The following supporting information can be downloaded at: https://www.mdpi.com/article/10.3390/polym16233250/s1.
