# Supplementary material for: Exploring Crystal Structure Features in Proton Exchange Membranes and Their Correlation with Proton and Heat Transport
Source: Polymers (Basel). 2024 Nov 22;16(23):3250. doi: 10.3390/polym16233250 (PMC11644780; doi:10.3390/polym16233250)
Supplement: Supplementary file 1 [file polymers-16-03250-s001.zip › polymers-3257717-supplementary.pdf]

Supporting Information

Exploring Crystal Structure Features in Proton Exchange Membranes and Their Correlation with Proton and Heat Transport

Cong Feng[a,*] , Cong Luo[a], Pingwen Ming[b], Cunman Zhang[b]

[a]College of Materials Science and Engineering, Tongji University, Shanghai 201804, China
[b]School of Automotive Studies, Tongji University, Shanghai 201804, China

Supplementary Note 1: Coarse-grained model of semicrystalline structure

One of novel aspects in this work is its modeling of the semicrystalline Nafion structure. Enough space is required for the corresponding model, i. e., to symbolize the amorphous molecular chains and many grains that make up semicrystalline structure. Here, the amorphous chain structure is added in a specific ratio after the crystal structure has been randomly distributed in the box. The amount of amorphous chain is determined according to the crystallinity.

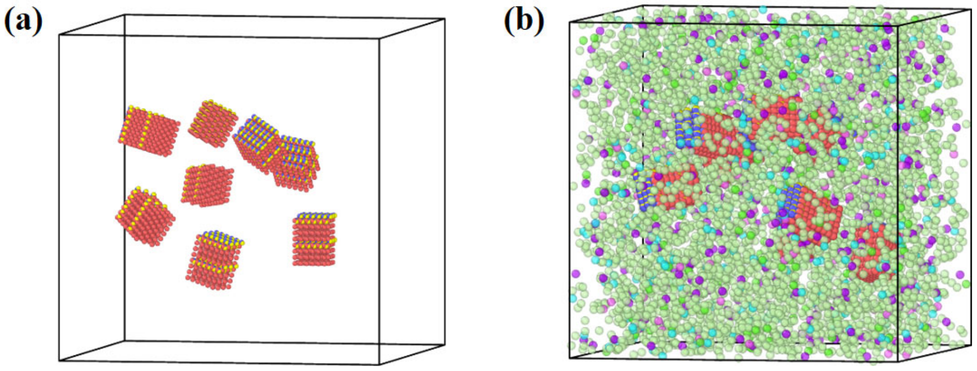

Figure S1. a) Crystal distributions. b) Coarse-grained model of semicrystalline structure

* Corresponding author. Email address: fengcong@tongji.edu.cn (C. Feng)

Supplementary Note 2: DREIDING force field and the corresponding parameters

The expressions of DREIDING force field used in our work is as follows:

$$E_{potential}=E_{bond}+E_{angle}+E_{torsion}+E_{improper}+E_{vdW}+E_{coul}+E_{hb} \quad \text{(S1)}$$

$$E_{bond}=K_{IJ}\left(r\text{-}r_{ij}\right)^2 \quad \text{(S2)}$$

$$E_{angle}=K_{IJK}[cos\,\theta\text{-}\,cos\,\theta_0]^2 \quad \text{(S3)}$$

$$E_{torsion}=V_{\phi}[1+cos\,(n\phi\text{-}d)] \quad \text{(S4)}$$

$$E_{improper}=\frac{1}{2}K(\frac{1}{sin\omega_0})^2\,(cos\omega\text{-}cos\omega_0)^2 \quad \text{(S5)}$$

$$E_{vdW}=4\varepsilon\left\{\left[\frac{\sigma}{r}\right]^{12}\text{-}\left[\frac{\sigma}{r}\right]^{6}\right\} \quad \text{(S6)}$$

$$E_{coul}=\frac{322.0637Q_iQ_j}{r_{ij}} \quad \text{(S7)}$$

$$E_{hb}=\varepsilon\left\{5\left[\frac{\sigma}{r}\right]^{12}\text{-}6\left[\frac{\sigma}{r}\right]^{10}\right\}cos^4\theta \quad \text{(S8)}$$

where $E_{potential}$, E_{bond}, E_{angle}, $E_{torsion}$, $E_{improper}$, E_{vdW}, E_{coul} and E_{hb} denote the total potential energy of the system, the bond energy, the bond angle energy, the dihedral angle energy, the out-of-plane vibrational energy, the van der Waals interaction energy, the electrostatic interaction energy and the hydrogen bonding energy, respectively. K_{IJ}, K_{IJK}, V_{ϕ} and K are elasticity constants, where the subscripts I, J, and K denote atoms I, J, and K, respectively, and ϕ represents the magnitude of the dihedral angle. r, θ, and ω represent the distance between atom pairs, the angle between atoms, and the angle of out-of-plane vibration, respectively. r_{ij} and θ_0 are the distance between atom i and atom j at equilibrium and the size of the bond angle at equilibrium, respectively. n and d are the dihedral angle constant and the integer value of the angle. ε and σ are the energy and distance parameters, respectively. Q_i and Q_j are the charges

of the atom *i* and atom *j*, respectively.

Table S1. Parameters in E_{bond}

Bond type	K_b (kcal·mol^{-1}·Å^{-2})	r_0 (Å)
C_3-C_3	350	1.53
C_3-F_	350	1.371
C_3-O_3	350	1.42
C_3-S_3	350	1.8
S_3-O_2	700	1.59
O_3-S_3	350	1.69
O_3-H_	350	0.98

Table S2. Parameters in E_{angle}

Angle type	K_θ (kcal·mol^{-1}·rad^{-2})	θ_0 (°)
C_3-C_3-F_	56.2498	109.471
F_-C_3-F_	56.2498	109.471
C_3-C_3-C_3	56.2498	109.471
C_3-C_3-O_3	56.2498	109.471
O_3-C_3-F_	56.2498	109.471
C_3-O_3-C_3	53.3490	104.51
C_3-C_3-S_3	56.2498	109.471
S_3-C_3-F_	56.2498	109.471
C_3-S_3-O_2	50.0672	92.1
C_3-S_3-O_3	50.0672	92.1
O_2-S_3-O_2	50.0672	92.1
O_2-S_3-O_3	50.0672	92.1

S_3-O_3-H_	53.3490	104.51

Table S3. Parameters in $E_{torsion}$

Dihedral angle type	K_{φ} (kcal·mol^{-1})	*n*	*d*
C_3-C_3-C_3-F_	1/9	3	0
F_-C_3-C_3-F_	1/9	3	0
C_3-C_3-C_3-C_3	1/9	3	0
C_3-C_3-C_3-O_3	1/9	3	0
F_-C_3-C_3-O_3	1/9	3	0
C_3-C_3-O_3-C_3	1/3	3	0
C_3-O_3-C_3-F_	1/3	3	0
O_3-C_3-C_3-O_3	1/9	3	0
O_3-C_3-C_3-S_3	1/9	3	0
F_-C_3-C_3-S_3	1/9	3	0
C_3-C_3-S_3-O_2	1/9	3	0
C_3-C_3-S_3-O_3	1/9	3	0
F_-C_3-S_3-O_2	1/9	3	0
F_-C_3-S_3-O_3	1/9	3	0
C_3-S_3-O_3-H_	1/3	2	0
O_2-S_3-O_3-H_	1/3	2	0

Table S4. Parameters in E_{vdw}

Atomic pair type	ε (kcal·mol^{-1})	σ (Å)
C_3-C_3	0.0951	3.473
C_3-O_3	0.0954	3.2531
C_3-S_3	0.1809	3.5317
C_3-O_2	0.0954	3.2531
C_3-F_	0.0830	3.2831
C_3-H_	0.0380	3.1597
O_3-O_3	0.0957	3.0332
O_3-S_3	0.1814	3.3117
O_3-O_2	0.0957	3.0332
O_3-F_	0.0833	3.0632
O_3-H_HB	0.0031	2.9398
S_3-S_3	0.3440	3.5903
S_3-O_2	0.1814	3.3117
S_3-F_	0.1579	3.3418
S_3-H_HB	0.00586	3.2184
O_2-O_2	0.0957	3.0332
O_2-F_	0.0833	3.0632
O_2-H_HB	0.0031	2.9398
F_-F_	0.0725	3.0932
F_-H_HB	0.0027	2.9698
H_-H_	0.0152	2.8464

The DREIDING force field included in the Materials Studio (MS) commercial software was compared with the force-field function and derived parameters mentioned above. Table S5 demonstrates the calculation results of a Nafion system with five Nafion chains and each chain with ten monomers. The consistency of the energy values in every component is evident, indicating the accuracy of the potential function we employed.

Table S5. Comparison of energy values

Energy (kcal·mol^{-1})	MS	Our model
$E_{potential}$	15363.071	15222.871
E_{bondl}	2415.244	2381.207
E_{anglel}	5096.777	5053.227
$E_{dihedriall}$	392.289	420.093
E_{vdW}	2469.539	2280.445
E_{coul}	4989.222	5087.899

Supplementary Note 3: governing equations of eDPD method and the specific parameters

Energy-conserving dissipative particle dynamics (eDPD) method allows the temperature of the system to change during the simulation[1]. The governing equations in eDPD simulation are as follows:

$$F_{ij}=F_{ij}^{C}+F_{ij}^{R}+F_{ij}^{D} \quad \text{(S9)}$$

$$q_{ij}=q_{ij}^{C}+q_{ij}^{V}+q_{ij}^{R} \quad \text{(S10)}$$

$$F_{ij}^{C}=(\frac{75k_BT}{\rho}+3.27\chi)\cdot\frac{T_i+T_j}{2}\left(1-\frac{r}{r_c}\right)\boldsymbol{e_{ij}} \tag{S11}$$

$$F_{ij}^{D}=-\gamma(1-\frac{r}{r_c})^{s}\left(\boldsymbol{e_{ij}}\cdot\boldsymbol{v_{ij}}\right)\boldsymbol{e_{ij}} \tag{S12}$$

$$F_{ij}^{R}=\sqrt{\frac{4\gamma k_BT_iT_j}{T_i+T_j}}(1-\frac{r}{r_c})^{\frac{s}{2}}\left(r_{ij}\right)\xi_{ij}\Delta t^{-\frac{1}{2}}\boldsymbol{e_{ij}} \tag{S13}$$

$$q_{ij}^{C}=C_v^2\kappa(T_i+T_j)^2/4k_B(1-\frac{r}{r_{CT}})^{sT}(\frac{1}{T_i}+\frac{1}{T_j}) \tag{S14}$$

$$q_{ij}^{V}=\frac{1}{2C_v}\left\{(1-\frac{r}{r_c})^{s}\left[\gamma_{ij}(e_{ij}\cdot v_{ij})^2\right]-\sigma_{ij}(1-\frac{r}{r_c})(\boldsymbol{e_{ij}}\cdot\boldsymbol{v_{ij}})\xi_{ij}\right\} \tag{S15}$$

$$q_{ij}^{R}=\sqrt{2k_Bk_{ij}}(1-\frac{r}{r_{CT}})^{\frac{sT}{2}}\left(r_{ij}\right)dt^{-\frac{1}{2}}\xi_{ij}^{e} \tag{S16}$$

where F_{ij}^C, F_{ij}^D and F_{ij}^R denote the conservative force, dissipative force and stochastic force, respectively. q_{ij}^C, q_{ij}^V, and q_{ij}^R stand for the collisional heat flux, viscous heat flux and stochastic heat flux, respectively. r is distance between particles i and j, $\boldsymbol{e_{ij}}$ the unit vector from particle j to i, and $\boldsymbol{v_{ij}} = \boldsymbol{v_i}-\boldsymbol{v_j}$ the velocity difference. k_B、T、ρ、and χ represent Boltzmann constant, temperature, number density and chi parameter, respectively. γ, s and ξ_{ij} are the dissipative force coefficient, temperature index and symmetric Gaussian random variable, respectively. C_v denotes the heat capacity. The mass of 4 water molecules (W) is taken as the unit mass (mW = 1), and the masses of the other beads are scaled accordingly. The radius Rc of the bead W is used as the unit length. The unit of energy is KT = 1. These three units are then used to represent the remaining units. The interactions between the beads are also calculated separately using the χ parameters based on the Flory-Huggins theory of polymer mixing. The corresponding parameters are given in Table S6 and Table S7.

Table S6. The χ parameter between different beads

	A	B	C	P	W
A	0	0.19416858	0.13142041	1.95325262	2.05359743
B		0	0.86340532	1.41165171	1.87160186
C			0	0.37090137	1.23993034
P				0	0.35177352
W					0

Table S7. Other main parameters in eDPD

Parameter	Value
ρ	4
γ	4.5
r_c	1.58
s	0.41+1.9 (T^2-1)
s_T	2
C_v (A, P, W)	36.11
C_v (B)	33.1
C_v (C)	30.09

Supplementary Note 4: critical simulation details

Mean square displacement curve is important to calculate the diffusion coefficient of the proton D, as shown in Fig. S2a. It must follow Einstein's diffusion law, which has a linear

relationship of log (MSD) and log (t). And the proton conductivity can then be calculated based on the relationship to *D*. Thermal transport behavior is investigated along the Z direction, as shown in Fig. S2b. The system is divided into two domains in the Z direction, with the upper domain being the +Q heat source and the lower domain being equally sized -Q heat sink. Therefore, there is a temperature gradient in the z direction (Fig. S2c), and the enthalpy and thermal diffusion coefficient can be calculated from information about the temperature distribution of the corresponding models.

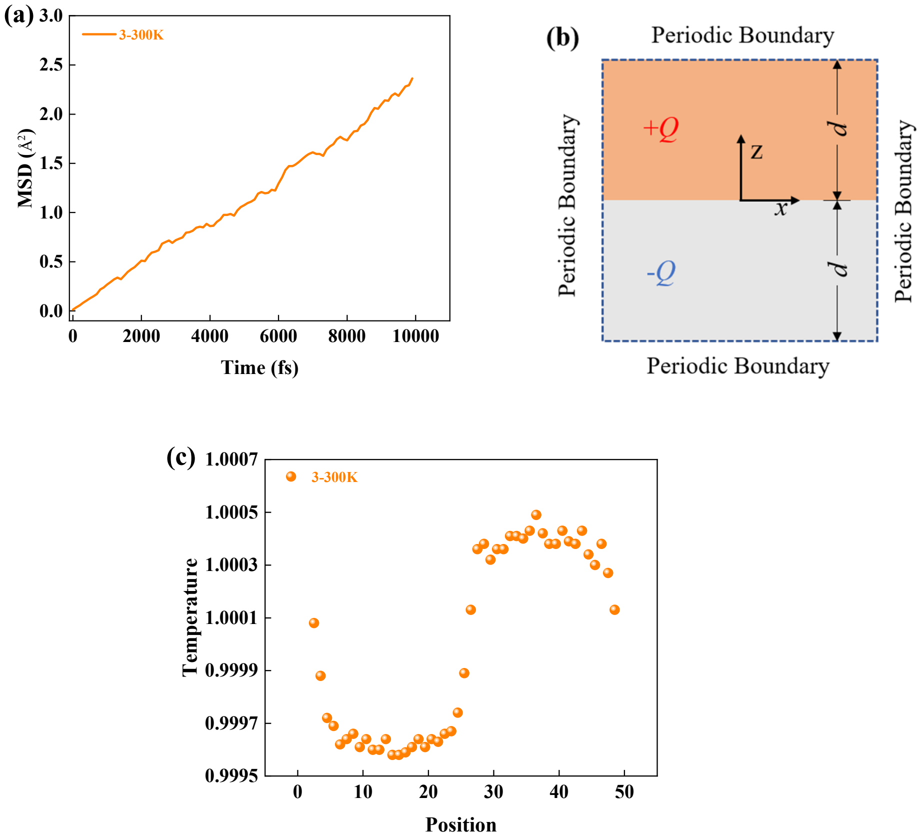

Figure S2. (a) MSD curve of a system with λ=3 at 300 K. (b) Schematic of thermal transport. (c) The temperature distribution along the Z-axis direction for a system with λ=3 at 300 K.

Supplementary Note 5: consistent water distribution results from AAMD and eDPD

simulations

Initially, two time and dimensional scales were used in AAMD and eDPD simulations to study the water distribution of crystal and amorphous structures. Fig. S3 illustrates how consistent the results of the two methods are at a given water content and temperature: the crystal structure displays more water channel connections than the amorphous structure, the water connections become smoother as the water content increases, and the temperature has less impact on the water connections.

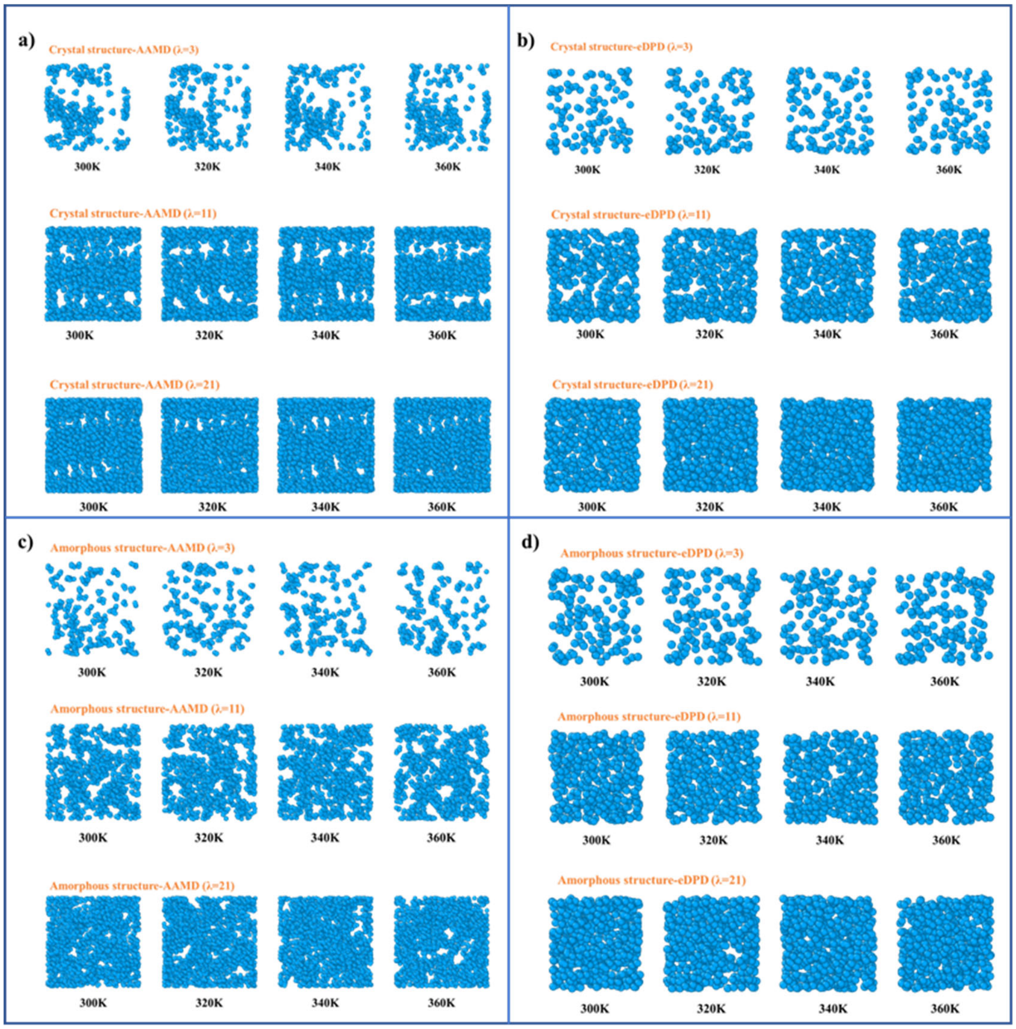

Figure S3. Water distribution in crystal structures of (a) all-atom and (b) coarse-grained

models. Water distribution in amorphous structures of (c) all-atom and (d) coarse-grained models.

Supplementary Note 6: comparison of σ_V and σ_E

Fig. S4a-4c displays a comparison of σ_V and σ_E, where σ_E values come from the literature[2] and σ_V are from our theoretical calculation. Fig. S4d summarizes the proton conductivities of semicrystalline structures (σ_V), which exhibit the same trend with temperature and water content as those in the case of amorphous and crystal structures, i.e., they show a positive correlation.

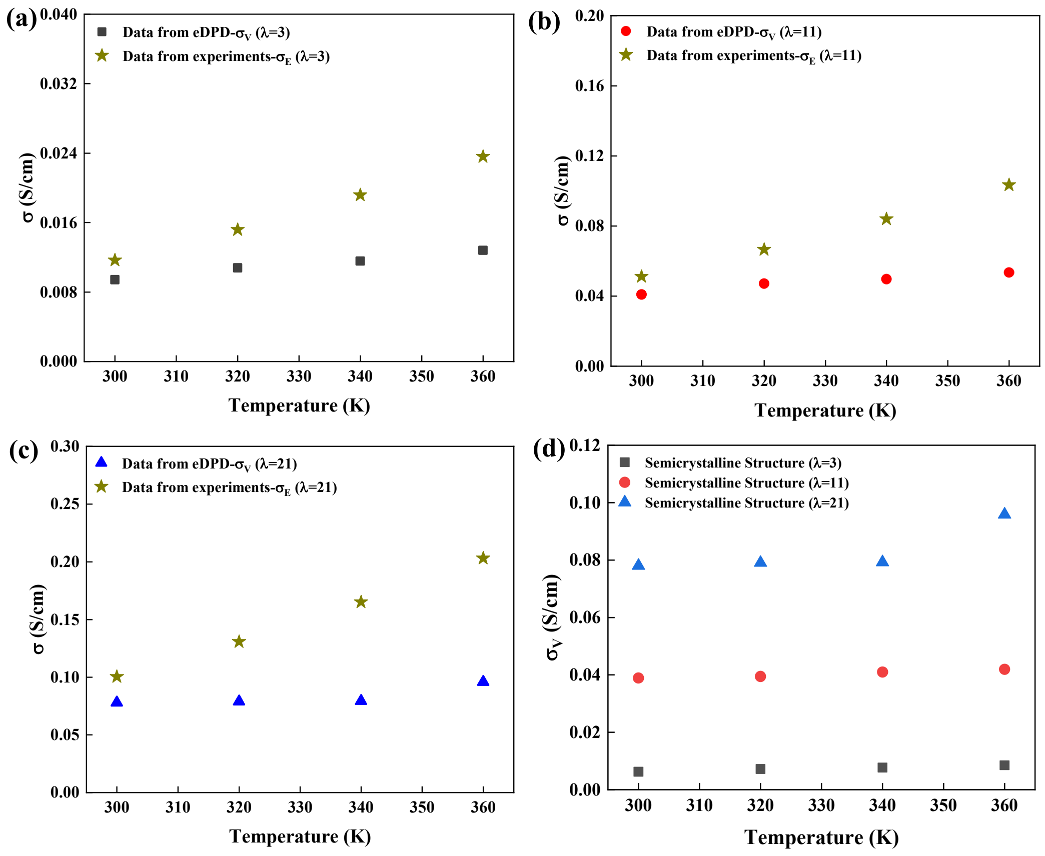

Figure S4. Comparison of proton conductivities from σ_V and σ_E at (a) λ = 3, (b) λ = 11 and (c) λ = 21. (d) σ_V values of semicrystalline structures.

Supplementary Note 7: atomic molar heat capacities

The accuracy of the thermal properties calculated by the eDPD method depends largely on the atomic molar heat capacities in the corresponding systems. Here we have used AAMD simulations to calculate these, as shown in Fig. S5. Interestingly, the molar heat capacity of the amorphous structure tends to increase with increasing hydration, whereas the molar heat capacity of the crystal structure is less affected by hydration. Overall, the molar heat capacity of these two structures does not change much under these environmental conditions.

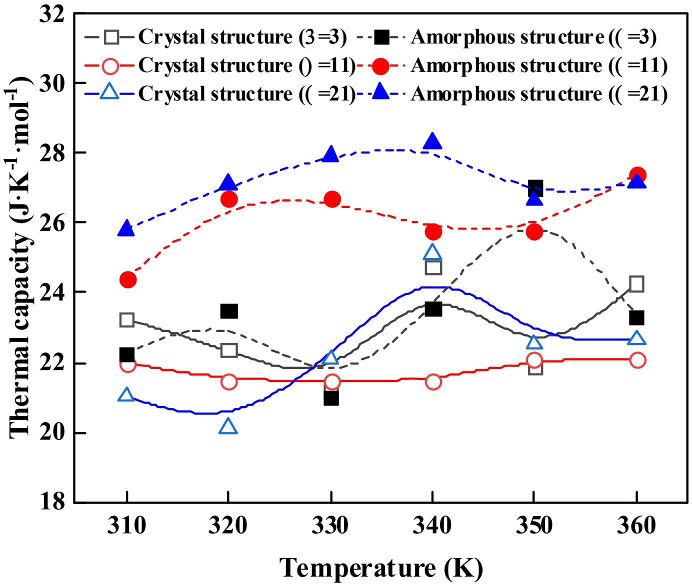

Figure S5. Atomic molar heat capacity of crystal and amorphous structures

References

[1] Z. Li, Y.H. Tang, H. Lei, B. Caswell, G.E. Karniadakis, Energy-conserving dissipative particle dynamics with temperature-dependent properties, J. Comput. Phys. 265 (2014) 113–127. https://doi.org/10.1016/j.jcp.2014.02.003.

[2] T.E. Springer, T.A. Zawodzinski, S. Gottesfeld, Polymer electrolyte fuel cell model, J.

Electrochem. Soc. 138 (1991) 2334–2342. https://doi.org/10.1149/1.2085971
